# Efficient Reprogramming of Naïve-Like Induced Pluripotent Stem Cells from Porcine Adipose-Derived Stem Cells with a Feeder-Independent and Serum-Free System

**DOI:** 10.1371/journal.pone.0085089

**Published:** 2014-01-20

**Authors:** Yu Zhang, Chao Wei, Pengfei Zhang, Xia Li, Tong Liu, Yong Pu, Yunsheng Li, Zubing Cao, Hongguo Cao, Ya Liu, Xiaorong Zhang, Yunhai Zhang

**Affiliations:** Anhui Provincial Laboratory for Local Livestock and Poultry Genetic Resource Conservation and Bio-breeding, Department of Animal Science, College of Animal Science and Technology, Anhui Agricultural University, Hefei, Anhui, People's Republic of China; Baylor College of Medicine, United States of America

## Abstract

Induced pluripotent stem cells (iPSCs) are somatic cells reprogrammed by ectopic expression of transcription factors or small molecule treatment, which resemble embryonic stem cells (ESCs). They hold great promise for improving the generation of genetically modified large animals. However, few porcine iPSCs (piPSCs) lines obtained currently can support development of cloned embryos. Here, we generated iPSCs from porcine adipose-derived stem cells (pADSCs), using drug-inducible expression of defined human factors (Oct4, Sox2, c-Myc and Klf4). Reprogramming of iPSCs from pADSCs was more efficient than from fibroblasts, regardless of using feeder-independent or feeder-dependent manners. By addition of Lif-2i medium containing mouse Lif, CHIR99021 and PD0325901 (Lif-2i), naïve-like piPSCs were obtained under feeder-independent and serum-free conditions. These successfully reprogrammed piPSCs were characterized by short cell cycle intervals, alkaline phosphatase (AP) staining, expression of Oct4, Sox2, Nanog, SSEA3 and SSEA4, and normal karyotypes. The resemblance of piPSCs to naïve ESCs was confirmed by their packed dome morphology, growth after single-cell dissociation, Lif-dependency, up-regulation of Stella and Eras, low expression levels of TRA-1-60, TRA-1-81 and MHC I and activation of both X chromosomes. Full reprogramming of naïve-like piPSCs was evaluated by the significant up-regulation of Lin28, Esrrb, Utf1 and Dppa5, differentiating into cell types of all three germ layers *in vitro* and *in vivo*. Furthermore, nuclear transfer embryos from naïve-like piPSCs could develop to blastocysts with improved quality. Thus, we provided an efficient protocol for generating naïve-like piPSCs from pADSCs in a feeder-independent and serum-free system with controlled regulation of exogenous genes, which may facilitate optimization of culture media and the production of transgenic pigs.

## Introduction

Embryonic stem cells (ESCs) are highly promising in regenerative medicine for their potential to differentiate into cells from all three embryonic germ layers. However, the application of ESCs is restricted, due to limited resource and ethical concerns [1]. Adult stem cells also can not be applied widely in clinics, as their limited capacity of differentiation. Since iPSCs, which share similar properties to ESCs [2], have been successfully derived from somatic cells with ectopic expression of transcription factors Oct4, Sox2, c-Myc and Klf4 [3], a new type of cells that avoids the abovementioned hurdles was provided. This may offer greater potential for *in vitro* disease modeling, drug screening and regenerative cell therapy. As the limited proliferative capacity and low frequency of homologous recombination of somatic cells, ESCs are regarded as a powerful cell resource for generating genetically modified animals, which can serve to increase our knowledge of mammalian physiology and disease [4]. Pig is often chosen as a research model for disease and regenerative medicine due to the close approximation of its body size, physical structure and metabolism to humans [5]. To date, *bona fide* ESCs have still not been established from domestic ungulates including pig. Hence, porcine iPSCs (piPSCs) have been thought as ideal substitutes for ESCs to produce genetically modified animals. However, difficulties remain in generating healthy offspring from piPSCs [4], and the quality of piPSCs may be the key factor affecting success of this aim.

Since piPSCs were first obtained by Wu et al [6], using a drug-inducible system with porcine ear fibroblasts and bone marrow mesenchymal stem cells (BMSCs), many piPSCs with different induction methods have been reported. Esteban *et al* successfully produced piPSCs from fibroblasts of Tibetan miniature pigs with retroviral vector [7]. Roberts *et al* described the piPSCs derived from porcine fetal fibroblasts by lentiviral transduction. However, of the two piPSC lines, one was found to have a chromosomal paracentric inversion [8]. Researchers then turned to obtaining piPSCs using episomes, while components of which were found to reside in the genome [9]. Subsequently, fibroblast-derived piPSCs induced with single retroviral plasmids consisting of mouse Oct4, Sox2, Klf4, and c-Myc were reported [10]. Thus, the starting cellular material for the generation of piPSCs is rare. Research from West *et al* showed that chimeric offspring could be obtained from porcine BMSC-derived iPSCs, the results are controversial since the use of PCR for identification only may not be sufficiently conclusive [11]. However, their work suggested that the cell type used for reprogramming can influence the development of iPSCs-derived embryos, and the source cell may impact the quality of piPSCs. Thus, evaluating new and appropriate types of cells from different sources is needed.

Porcine iPSCs, which generated with different methods, have been used to inject into enucleate oocytes to produce offspring by nuclear transfer [4]. These piglets can be obtained only from iPSCs created by drug-inducible system, suggesting that the method of generating iPSCs has a prominent role in successfully producing porcine offspring. However, all cloned piglets using piPSCs as donors died after birth, indicating quality of iPSCs is unsatisfied. Hence, the quality of piPSCs is required to be promoted by exploring more appropriate reprogramming protocols.

In general, ESCs are derived traditionally from the inner cell mass (ICM) of preimplantation blastocysts. Recently, a new type of pluripotent stem cells (PSCs) was established from post-implantation epiblasts, which was named Epiblast stem cells [12]. They are believed to be of different pluripotent states: naïve and primed, respectively. Primed and naïve PSCs share some core features, such as expression of genes associated with pluripotency, as well as the ability to differentiate into cells from three germ layers *in vitro* and *in vivo* [13], [14]. However, they are clearly distinct from each other in many aspects such as morphology, gene expression, developmental capacity and cytokines to maintain their undifferentiated state [15]. For primed PSCs, characteristics similar to those of human ESCs are observed, such as a flattened colony morphology [16], a difficulty in management after single-cell dissociation, the requirement of basic fibroblast growth factor (bFGF) and transforming growth factor (TGF) β/activin A signaling for self-renewal [12], [17], and have only one active X chromosome in female cells (XiXa). In contrast, naïve PSCs present a packed dome morphology [18], [19], and their colonies can be formed with high efficiency after single-cell dissociation. Furthermore, JAK/STAT3 signalling is sufficient for the maintenance of naïve pluripotency [15], [20], and always undergo X-chromosome activation (XaXa), the specific expression of Eras and Stella have been also used to identify the naïve PSCs [16], [21]. Compared to primed PSCs, naïve PSCs are more efficient in repopulating the ICM upon aggregation or injection into host blastocysts [22]. It has been claimed that naïve-like piPSCs had been established from embryonic fibroblasts. However, no viable offspring of chimera or nuclear transfer was reported from them, and this might be due to incomplete reprogramming and induction method, which resulted in immature teratomas' formation and sustained transgenes' expression [21]. Thus, the completely reprogrammed piPSCs of naïve-like state is a hopeful and potential material to benefit the production of iPSCs-derived offspring.

Traditional reprogramming environment for generation of iPSCs usually contains animal product-based components, such as mouse feeder cells and bovine serum in particular, which are important for the process [23]. Due to the lack of fully understanding of cell culture requirements, bovine serum is always supplied in the various culture media, providing a range of growth factors and nutrients to stimulate the growth, differentiation and attachment of cells [24]. It has been shown that the secretion of Lif, fibroblast growth factor 2 (FGF2), TGF ß1 from mouse feeder is known to be essential for the maintenance of generated iPSCs [25]. However, the use of bovine serum and mouse feeder cells are hurdles in clinical applications, as the undefined components of the system may cause variabilities [23], which might compromise the development of iPSCs-derived embryos. Feeder-independent and serum-free culture systems have been reported for human and mouse ESCs [26], [27], [28], while it is rare for piPSCs [10]. Consequently, to explore whether serum-free and feeder-independent system can be integrated into piPSCs generation system is of great need and practical value for the researches of PSCs in large animals.

Thus, our goal of the present study is to establish an improved method for generating naïve-like piPSCs from a more accessible source in serum-free and feeder-independent conditions, and a new resource material obtained here is expected to facilitate the production of iPSC-derived porcine offspring.

## Materials and Methods

### Ethics Statement

The pig specimens were purchased from Anhui HUAAO pig breeding Co., Ltd., a branch of HUAAO Group. And they permitted the porcine parts to be used for the scientific and educational research in our lab. The treatment of porcine samples was based on the protocol of the farm animal research guidelines approved by the Animal Research Committee of Anhui Agricultural University. All animal procedures were approved by the Animal Health Care Committee of Anhui Agricultural University.

### Chemicals

All chemicals were purchased from Sigma (USA) unless otherwise stated.

### Isolation, Culture of Porcine Adipose Derived Stem Cells (pADSCs) and Ear Fibroblasts (pEFs)

Subcutaneous adipose tissue was obtained from the 28-day-old Danish Landrace female piglets, and enzymatically dissociated in Dulbecco's modified Eagle medium/F12 (DMEM/F12) with 0.09% collagenase type I and 10% fetal bovine serum (FBS, Life Technologies, USA). After incubating in 37°C for 90 min, the dissociation was terminated by washing medium (DMEM/F12 with 10% FBS), followed by centrifugation for 5 min (320×g). Pellet was resuspended with washing medium, and sequentially filtered through 250 µm, 80 µm and 25 µm nylon mesh to remove the tissue debris. Being washed for three times, cells were suspended with DMEM/F12 containing 10% FBS, 50 µg/ml vitamin C and 10 ng/ml basic fibroblast growth factor (bFGF, Pepro Tech, USA), and cultured at 37°C, 5% CO_2_ in a humidified incubator. Culture medium was changed every three days until reaching 80%–90% confluency.

Ear tissue of same piglets was washed with DPBS, after the capillus were scraped, marginal tissue were cut into pieces with 3 cm×3 cm. Explants were transferred into 10 cm dish and humidified with serum. Dish was inverted and cultured at 37°C, 5% CO_2_ for 8 h. Then culture medium (DMEM with 15% FBS) were added into the upright dish with a final volume of 8 ml. When cells reached 80%∼90% confluence, they were trypsinized and cryopreserved.

### Identification of pADSCs

The identification of pADSCs was performed following the guidelines of the International Society for Cellular Therapy [29]. Proliferative ability was evaluated by monitoring the growth curve and sequential passaging of cells. At 80–90% confluency, pADSCs were passaged by trypsinization, and re-seeded at an initial concentration of 10,000 cells/well in a 24-well plate. Three wells per plate were counted every 24 h, and pADSCs were continuously passaged until replicative senescence was observed.

The differentiation potential of pADSCs was estimated by adipogenesis and osteogenesis, they were performed according to the manufacturer's protocols of the Human Mesenchymal Stem Cell Adipogenic Differentiation Medium and Human Mesenchymal Stem Cell Osteogenic Differentiation Medium (both from Cyagen Biosciences, USA). Mature adipocytes were detected by Oil Red O staining, and osteogenesis was confirmed by staining mineralized nodules of the differentiated cells with Alizarin Red S.

Typical surface markers of pADSCs were analyzed by flow cytometry. When the pADSCs reached 80%–90% confluency at passage 3, they were dissociated using TrypLE (Life Technologies, USA), and incubated with FITC-conjugated, PE-conjugated and AF647-conjugated monoclonal antibodies, which directed toward CD29 (BD Pharmingen, USA), CD44 (eBioscience, USA), CD90 (BD Pharmingen, USA), CD105 (BD Pharmingen, USA), CD45 (eBioscience, USA), CD31 (eBioscience, USA), and HLA-D/DR/DQ (Santa Cruz, USA). Non-specific binding of antibodies was determined by isotype controls (ISO). After 30 min of incubation, cells were washed with cold Dulbecco's phosphate-buffered saline (DPBS) three times, and resuspended in 1% paraformaldehyde at a density of 1×10^6^ cells/ml. Then they were analyzed by the flow cytometry (BD FACS Calibur, USA). Data were analyzed using FlowJo software (version 7.61).

### Lentiviral Production and Transduction

293T cells (Life Technologies, USA) were grown to 90% confluency and transfected with 3.6 µg of each lentiviral vector (Oct4, Sox2, Klf4, c-MYC) plus 1.8 µg Pvsvg and 2.7 µg delta 8.91 per T-25 flask using Fugene HD (Roche, USA). The culture medium was replaced with 4 ml fresh medium 24 h later, and lentiviral supernatant was harvested 48 h after transfection, and centrifuged at 800×g for 5 min to pellet the cellular debris. The lentiviral-containing supernatant was transferred and stored at 2∼8°C in a week.

Porcine ADSCs and porcine ear fibroblasts (pEFs) were plated at a density of 2,500 cells/cm^2^ in feeder-containing and feeder-independent condition, and were incubated with doxycycline (DOX)-inducible lentivirals carrying reprogramming factors (Oct4, Sox2, c-Myc and Klf4). 48 h later, cells were passaged onto matrigel (BD Biosciences, USA) coated plates with inactivated mouse embryonic fibroblast (MEF) feeders (Sidansai, China) from CF-1 mice, and cultured with growth medium for additional 24 h. Subsequently, growth medium was replaced with DOX (clontech, USA) only medium for 5 days, which consisted of DMEM/F12, 20% knockout serum replacement (KSR, Life Technologies, USA), 2 mM L-glutamine (Life Technologies, USA), 0.1 mM non-essential amino acids (Life Technologies, USA), 0.1 mM β-mercaptoethanol (Life Technologies, USA) and 4 µg/ml DOX. Thereafter, the DOX only medium was removed completely, and the Lif-2i medium was added into the plated for another 2 days, which consisted of DMEM/F12, 15% KSR, GlutaMAX™-1 (Life Technologies, USA), 0.1 mM β-mercaptoethanol, N2 (Life Technologies, USA), B-27 (Life Technologies, USA), PD0325901 (Selleck, USA), CHIR99021 (Selleck, USA), mouse Lif (Millipore, USA) and 4 µg/ml DOX. Finally, iPSCs colonies were picked and maintained in Lif-2i medium for the following culture. For passaging, they were dissociated using TrypLE (Life Technologies, USA) every 2 to 3 days.

### Alkaline Phosphatase (AP) Staining and Immunofluorescence Staining

AP staining was performed according to the manufacturer's (Sidansai, China) instructions. For the immunofluorescence staining, cells were fixed with 4% formaldehyde in DPBS for 15 min, permeabilized with 1% Triton X-100 in DPBS for 15 min, and blocked with 2% bovine serum albumin in DPBS for 1 h. Thereafter, cells were incubated with primary antibodies for 1 h, including those antibodies Oct4 (1∶200, Abcam), Sox2 (1∶200, Cell Signaling), Nanog (1∶200, Abcam), TRA-1-60 (Millipore, 1∶200), TRA-1-81 (Millipore, 1∶200), SSEA1 (1∶50, Developmental Studies Hybridoma Bank), SSEA3 (1∶50, Developmental Studies Hybridoma Bank), SSEA4 (1∶50, Developmental Studies Hybridoma Bank), 5-methyl cytidine (5-mC, 1∶200, Abcam), 5-hydroxymethyl cytidine (5-hmC, 1∶200, Active Motif), H3K27me3 (1∶250, Millipore). Primary antibodies were detected using secondary antibodies conjugated to Alexa Fluor 488 (1∶500, Molecular Probes) and Alexa Fluor 594 (1∶500, Molecular Probes).

### Real-time PCR and Reverse Transcription PCR

Total RNA and cDNA of each sample were prepared using the RNeasy Mini kit (Qiagen, Germany) and the QuantiTect Reverse Transcription kit (Qiagen, Germany) according to the manufacturer protocols. Real-time PCR was performed in a StepOnePlus Real-time PCR System (Applied Biosystems, USA). The FastStart SYBR Green Master mix (Roche, USA) was used for PCR, and GAPDH was chosen as an endogenous control. cDNA samples were subjected to PCR amplification with primers for the GAPDH, Endo Oct4, Endo Sox2, Endo c-Myc, Endo Klf4, Nanog, Lin28, Dnmt3b,Tert, Esrrb, Utf1, Dppa5, Stella and Eras (Table S1). For the Reverse transcription PCR, cDNA samples were subjected to Neurod, Sox9, Pdx1, Exo Oct4, Exo Sox2, Exo c-Myc, Exo Klf4 and GAPDH (Table S2).

### Karyotype Analysis

Karyotyping was performed at the Xiangtan Center Hospital using standard protocols for high-resolution G-banding.

### Bisulfite Sequencing

Genomic DNA (1 µg) samples extracted from naïve-like piPSCs and pADSCs were treated with bisulfite using a CpGenome modification kit (Chemicon, USA) according to the manufacturer's protocol. The treated samples then were subjected to semi-nested PCR with Nanog primers (Table S3). Purified PCR products were cloned into a T-vector (TIANGEN, China), and ten randomly selected colonies were individually sequenced.

### Embryoid Body Formation

Naïve-like piPSCs were treated with TrypLE, and transferred to ultra-low attachment plates in suspension culture for 9 days with DMEM/F12 containing 20% KSR, 2 mM L-glutamine, 0.1 mM non-essential amino acids and 0.1 mM β-mercaptoethanol. The total RNA of embryoid bodies (EBs) was extracted for the Reverse Transcription PCR analysis.

### Teratoma Formation

The naïve-like piPSCs (5×10^6^) were injected into non-obese diabetic/severe combined immune deficient (NOD/SCID) mice. Four months after the injection, tumors were harvested, dissected, fixed in 4% paraformaldehyde and processed for hematoxylin-eosin (H&E) staining.

### Nuclear Transfer Embryos Production and Culture

For construction of piPSCs nuclear transfer embryos, in vitro matured porcine oocytes according to previous report [5] were fixed by holding pipette on an inverted microscope (Olympus, Japan) equipped with a micromanipulator (Narishige, Japan) and warmed stage (Tokihai, Japan). Then oocytes were enucleated by removing the first polar body aspirated out by denucleation/injection pipette together with 10%–20% of the adjacent cytoplasm, presumably containing the metaphase plate. A selected donor cell that was globular, smooth, strongly refractive was injected subsequently into the perivitelline space through the same slot. After the manipulation, reconstructed donor cell - oocyte cytoplasm couplets were then transferred into T2 drops compose of HEPES - buffered TCM199 plus 2% (v/v) FBS, incubated at 38.5°C, 5% CO_2_ in a humidified incubator for 30 min.

For fusion and activation, the reconstructed couplets, which had been recovering, were transferred in batches into the fusion chamber filled with the fusion/activation liquid (0.3 M Mannitol, 0.1 mM CaCl_2_, 0.1 mM MgCl_2_, 0.5 mM HEPES and 0.01% PVA). The couplets were aligned gently such that the interface of the donor cells and acceptor oocytes were parallel to electrodes, then fused and activated using a electrofusion machine (BLS, Hungary). Subsequently, couplets were washed three times in embryo culture medium PZM-3 and transferred into chemically assisted activation liquid (PZM-3 plus 10 µg/ml cycloheximide and 10 µg/ml cytochalasin B) covered with mineral oil and incubated. Four hours later, fusion results were examined under a stereomicroscope and fused embryos were placed into a drop of pre-equilibrated culture media at a density of 15 per 50 µL, and were cultured at 38.5°C, 5% CO_2_ in a humidified incubator. Embryo cleavage and blastocyst development were observed and documented at day 2 and 6, respectively.

### Fluorecent Staining of Blastocysts

Nuclear staining was performed for counting the total nuclei number of blastocysts. Briefly, day 6 blastocysts were fixed for 10 min in DPBS containing 4% paraformaldehyde, mounted on clean glass slides, and stained with a glycerol - based DAPI (2 µg/ml) solution for 10 min at room temperature in darkness. Total number of stained nuclei, which appeared blue when visualized under UV illumination of an inverted fluorescence microscope (Olympus, Japan) fitted with blue filter, were then counted for individual blastocysts, and digital images were taken.

### Statistical Analysis

Data are presented as mean ± S.E.M. values, differences between groups were evaluated using SPSS17.0 (IBM, USA). Statistical significance was considered for *P* values <0.05.

## Results

### Characterization of pADSCs

**Figure 1:**
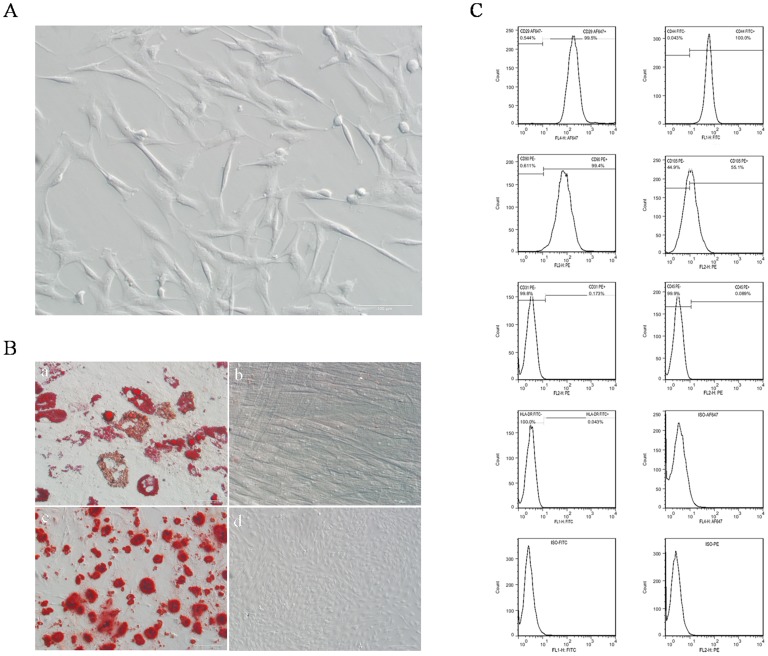
Identification of pADSCs. (A) Morphology of pADSCs at passage 3, scale bar = 100 µm. (B) Multi-lineage differentiation of pADSCs, mature adipocytes were detected by Oil Red O staining (a), scale bar = 50 µm; osteogenesis was analysis by Alizarin Red S staining (c), scale bar = 200 µm. Cells cultured in the corresponding proliferation medium served as negative controls, respectively (b, scale bar = 50 µm; d, scale bar = 200 µm). (C) Expression of cell surface markers in pADSCs at passage 3 including CD29, CD44, CD90, CD105,CD31, CD45 and HLA-DR. Positive cells were gated based on staining with isotype antibody controls.

Approximately 30–50 ml of porcine subcutaneous adipose tissue was obtained from one piglet. Porcine ADSCs isolated from the tissue showed a spindle-like fibroblastic morphology (Fig.1A), and could proliferate rapidly (doubling time = 21.09 h) in low passage (Fig S1), which managing to undergo 20 passages without significant replication senescence. After adipogenesis and osteogenesis of pADSCs for a certain period *in vitro*, mature adipocytes and mineralized calcium deposition were observed by Oil Red O and Alizarin Red S staining, respectively (Fig. 1B). The analysis of surface markers on pADSCs demonstrated the isolated cells expressed high level of CD29 (0.995±0.0577), CD44 (0.999±0.0333), CD90 (0.994±0.0333) and CD105 (0.550±0.122), and negative for the CD31 (0.0016±0.00013), CD45 (0.000793±0.0000689) and HLA-DR (0.00051±0.00008) (Fig. 1C).

### Efficient Reprogramming of Naïve-like piPSCs from pADSCs

Firstly, the exact period of effect of Lif-2i medium on reprogramming of the cells was investigated. In our preliminary experiment, we replaced the DOX-only media with Lif-2i media 1, 3 and 5 days after the onset of DOX-only media culture (day 0). Our results showed that when the Lif-2i medium was added 1 day later, the transduced cells failed to expand and entered apoptosis during further culture. And if the Lif-2i media were employed at day 3, colony-like cells could be observed, and they would differentiate gradually at day 7. In contrast, when using the Lif-2i to replace the used media at day 5, many colonies with the typical morphology of mouse ESCs formed. Therefore, in the following experiments, lentiviral transduced cells were treated in DOX only medium for 5 days, and then the Lif-2i media will be employed afterwards for further reprogramming.

**Figure 2:**
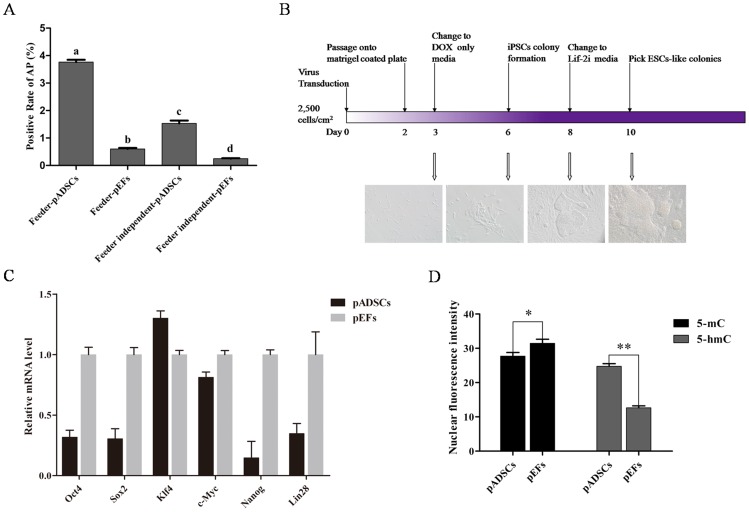
Generation of piPSCs from pADSCs. (A) AP staining of colonies which were reprogrammed from pADSCs and pEFs in the presence of feeders and feeder-independent condition. The rate of AP positive colonies was compared between the groups, different superscripts above the bars denote significant difference (*P*<0.05). (B) Schematic of the reprogramming strategy and the, morphology of cells at day 3, 6, 8 and 10. (C) Expression levels of genes associated with reprogramming, including Oct4, Sox2, c-Myc, Klf4, Lin28 and Nanog were evaluated in pADSCs and pEFs. (D) Expression levels of 5-mC and 5-hmC were analyzed in pADSCs and pEFs by immunofluorescence staining, *P<0.05, **P<0.01.

To demonstrate the efficient reprogramming of pADSCs, we initially compared the piPSCs generation efficiency from pADSCs and pEFs on feeder condition. Lentiviral transduced cells were plated at 5,000 cells/cm^2^ on feeder layers. However, large numbers of small colonies were observed 5 days after DOX addition from the both source cells, which interfered with further reprogramming (data not shown). Thus, the transduced cells were plated at 2,500 cells/cm^2^ on feeder layers for the next experiment. After 7 days of reprogramming, an approximately six-fold higher number of AP positive colonies was observed in the pADSC group (0.0376±0.000814) compared to the pEF group (0.00601±0.000393) (Fig. 2A).

We then compared the efficiency of generating piPSCs from pADSCs and pEFs in feeder-independent condition. Lentiviral transduced cells were plated at 2,500 cells/cm^2^ on matrigel coated dishes. These cells exhibited a spindle-like morphology 24 h later (day 3), and small loose colonies formed after next 3 days (day 6). Prior to the change of Lif-2i medium, many colonies were displaying human ESC-like morphology. After two days of treatment with Lif-2i medium, they exhibited a packed and slightly domed state (Fig. 2B). As for the reprogramming efficiency, the rate of AP positive colonies in the pADSC (0.0153±0.00106) group was significantly higher than that in the pEF group (0.00247±0.000196). For both pADSCs and pEFs, the reprogramming process on feeder layers was more efficient than that in feeder-independent conditions (Fig. 2A). Due to the benefits of cellular reprogramming in feeder-independent conditions, further studies were focused on this type of system. Porcine iPSCs with naïve-like state were finally obtained, and two lines of them were characterized fully in present study, which were designated as C4-6 NpiPSCs and C4-30 NpiPSCs.

Nextly, intrinsic expression levels of key reprogramming factors in pADSCs and pEFs, including Oct4, Sox2, Klf4, c-Myc, Nanog and Lin28 were analyzed, in parallel to the epigenetic status of the two cell lines. We found that, except for Klf4, the expression levels of Oct4, Sox2, c-Myc, Nanog and Lin28 were lower in pADSCs than those in pEFs (Fig. 2C). Therefore, the whole genomic methylation and demethylation levels of pADSCs and pEFs were evaluated through 5-mC and 5-hmC detection (Fig S2). Results of the analysis showed that the level of 5-hmC in pADSCs was higher than that in pEFs, meanwhile the level of 5-mC in pADSCs was lower than that in pEFs (Fig. 2D).

### Identification of NpiPSCs

**Figure 3:**
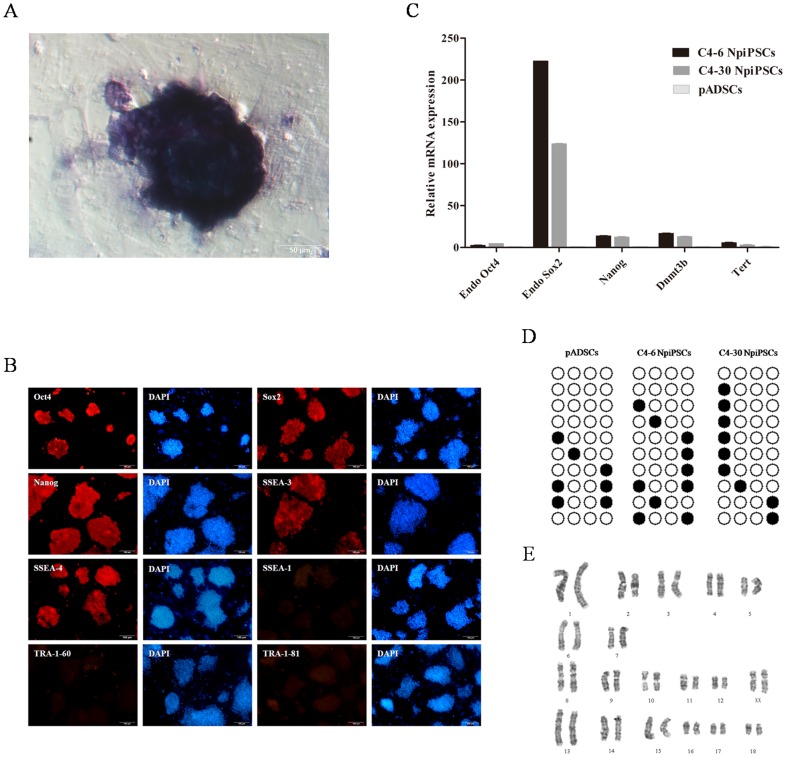
Characterization of naïve-like piPSCs produced by DOX-inducible system. (A) AP staining of naïve-like piPSCs, scale bar = 50 µm. (B) Pluripotency of naïve-like piPSCs was demonstrated by immunofluorescence staining of Sox2, Nanog, SSEA1, SSEA3, SSEA4, TRA-1-60 and TRA-1-81, scale bar = 100 µm. (C) Real-time PCR analysis of expression level of pluripotency genes in pADSCs, C4-6 NpiPSCs and C4-30 NpiPSCs. (D) DNA methylation analysis of the Nanog promoter in C4-6 NpiPSCs and C4-30 NpiPSCs. (E) Karyotype analysis of naïve-like piPSCs.

Colonies were picked out and serially expanded rapidly on irradiated MEF feeders for more than 30 passages. Similar to mouse ESCs, NpiPSCs were positive for AP (Fig.3A). The analysis of immunofluorescence staining has confirmed the expression of pluripotency markers in these piPSCs, including Oct4, Sox2, Nanog, SSEA3 and SSEA4, while they were negative for SSEA1 (Fig.3B). Furthermore, genes associated with puripotency, including Endo Oct4, Endo Sox2, Nanog, Dnmt3b and Tert, were markedly elevated compared with those in parental pADSCs (Fig.3C). The epigenetic status of the Nanog promoter was analyzed by bisulfite genomic sequencing, which showed that it was highly unmethylated in NpiPSCs. However, CpG dinucleotides in these regions also were highly unmethylated in the parental pADSCs (Fig. 3D). Both of the NpiPSC lines subjected to cytogenetic analysis possessed a normal karyotype of 38XX at passage 12 (Fig. 3E).

### Evidences of NpiPSCs in a Naïve-like State

**Figure 4:**
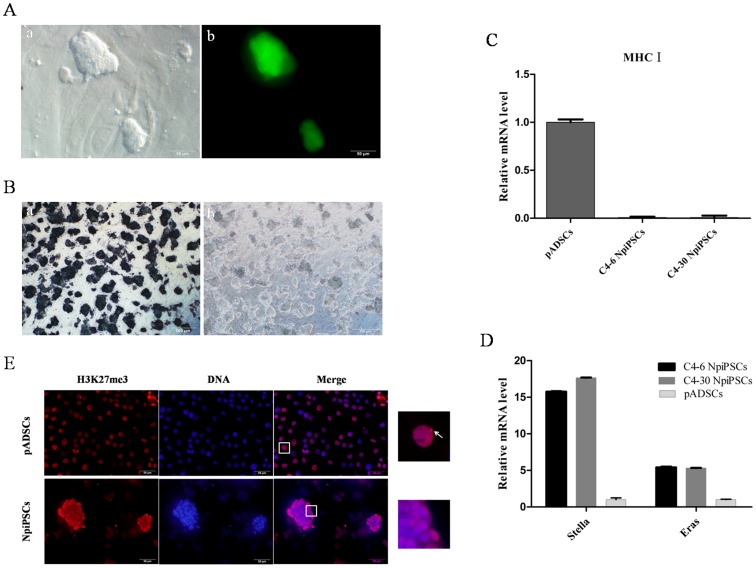
Evidences of naïve-like state of piPSCs. (A) Morphology of naïve-like piPSCs at phase contrast (a, scale bar = 50 µm) and immunofluorescence (GFP) imaging (b, scale bar = 50 µm). (B) AP staining of C4-6 NpiPSCs, when Lif was present (a, scale bar = 500 µm) and withdrawn (b, scale bar = 500 µm). (C) mRNA levels of MHC I in pADSCs, C4-6 NpiPSCs and C4-30 NpiPSCs. (D) mRNA levels of stella and Eras in pADSCs, C4-6 NpiPSCs and C4-30 NpiPSCs. (E) Immunofluorescence staining of H3K27me3 in pADSCs and naïve-like piPSCs (C4-30 NpiPSCs), nuclei surrounded by squares were magnified (right panels), arrows indicate H3K27me3-positive areas, scale bar = 50 µm.

Both of the C4-6 NpiPSCs and C4-30 NpiPSCs exhibited a packed dome morphology, which were similar to mouse ESCs (Fig. 4A), and a few dead cells could be observed after dissociation into single cells for passaging or cryopreservation (data not shown). New colonies soon formed and further passaging was needed within 48 h after passaging, due to the short doubling time of the two cell lines (16.3±0.0309 h and 15.6±15.809 h for C4-6 NpiPSCs and C4-30 NpiPSCs, respectively). And they were strongly indispensable for the Lif, in case the Lif was withdrawn, AP expression would disappear (Fig.4B). Consistent with the generally low levels of major histocompatibility complex I (MHC I) expression in ADSCs, no obvious expression of MHC I could be detected in C4-6 NpiPSCs and C4-30 NpiPSCs (Fig. 4C). Moreover, Stella and Eras were highly up-regulated in these two cell lines (Fig. 4D). The activation of X chromosome in cells was estimated by the immunofluorescence staining of H3K27me3 foci in nuclear. Apparently, H3K27me3 foci was found in pADSCs, while it was not observed in naïve-like piPSCs (Fig. 4E).

### Evidences of Full Reprogramming of NpiPSCs

**Figure 5:**
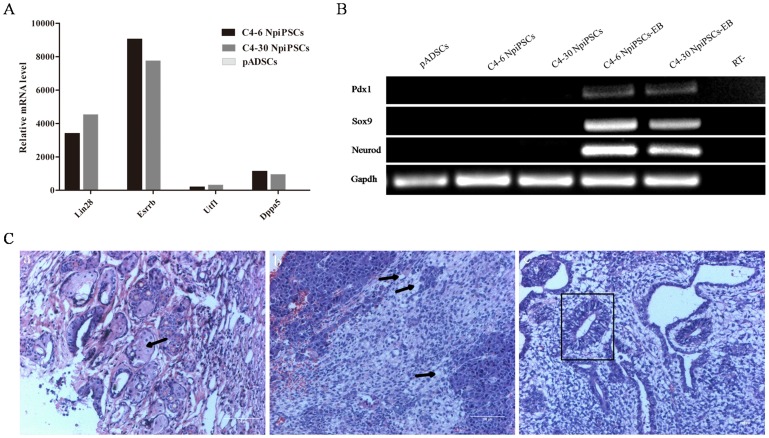
Characterization of fully reprogrammed porcine naïve-like iPSCs. (A) Real-time PCR analysis of expression levels of genes associated with fully reprogramming in pADSCs, C4-6 NpiPSCs and C4-30 NpiPSCs. (B) Reverse transcription PCR analysis of differentiation markers for the three germ layers in the EBs. (C) Hematoxylin and eosin staining of naïve-like piPSCs-derived teratoma (C4-30 NpiPSCs), the tumor was differentiated into the tissues of three germ layers, including cuticulated epithelium (a, ectoderm), adipose tissue (b, mesoderm) and gut-like epithelium (c, endoderm). Black arrows and square denote the specific structure of ectoderm mesoderm and endoderm, scale bar = 100 µm.

Genes related to fully reprogramming including Lin28, Esrrb, Utf1 and Dppa5 were evaluated and found to all be significantly elevated in the NpiPSCs (Fig.5A). To estimate their capacity of differentiation, we examined the differentiation potential of NpiPSCs by embryoid body (EB) and teratoma formation. For the *in vitro* differentiation, typical EBs were harvested after 9 days of suspension culture. Successful differentiation was confirmed by Reverse Transcription PCR analysis of the markers of three germ layers, which showed that they were positive for Neurod (ectoderm), Sox9 (mesoderm) and Pdx1 (endoderm), while these genes were negative in undifferentiated pADSCs and NpiPSCs (Fig. 5B).

We next addressed whether the NpiPSCs could directly differentiate into cell lineages of the three germ layers *in vivo*. Teratomas were formed 4 months later after injection of NpiPSCs into NOD/SCID mice. Histological analysis of the teratomas revealed three germ layers in the tissues, including cuticulated epithelium (ectoderm), adipose tissue (mesoderm) and gut-like epithelium (endoderm) (Fig.5C). These results showed that the NpiPSCs possess similar *in vitro* and *in vivo* differentiation abilities to those of mouse ESCs.

### NpiPSCs for Nuclear Transfer

**Figure 6:**
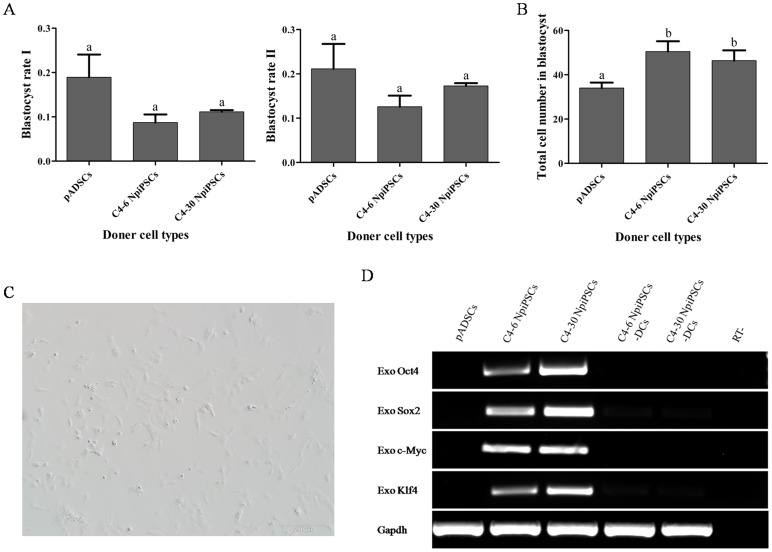
NpiPSCs for nuclear transfer. (A) Blastocyst rate of nuclear transfer embryos from NpiPSCs and pADSCs. Blastocyst rate I  =  No. blastocyst/No. cultured embryos (a); Blastocyst rate II  =  No. blastocyst/No. cleaved embryos (b). Different superscripts above the bars denote significant difference (P<0.05). (B) Total cell number of blastocyst from NpiPSCs and pADSCs. (C) Spindle-like morphology of cells 24h after the withdrawal of DOX. (D) Reverse transcription PCR analysis of expression of exogenous genes 6 days after the withdrawal of DOX.

No significant difference were observed between pADSCs and NpiPSCs based nuclear transfer embryos for the rate of blastocyst (Fig. 6A), while the total cell number of blastocyst from NpiPSCs was significantly higher than that from pADSCs (Fig. 6B). In addition to support reconstructed embryo development *in vitro*, it was confirmed that NpiPSCs would soon differentiate into cells with heterogeneous morphology with 24 hours after the DOX was withdrawn, including small spindle-like fibroblastic and dot morphologies (Fig. 6C). Six days later, the expression of exogenous genes was nearly completely silent (Fig. 6D).

## Discussion

In this study, we successfully established porcine iPSCs from pADSCs in a feeder-independent and serum-free system, the process of which was more efficient than that from pEFs. Features of the reprogrammed cells satisfy criteria of multipotency and a naïve-like state. In particular, the differentiation capacity of these NpiPSC cell lines in a naïve-like state and their regulatable silencing of exogenous genes render them potentially useful tools for the generation of porcine offspring.

To improve the quality and quantity of human and mouse iPSCs, many studies have been focused on cellular material for reprogramming, including neural stem cells (NSCs) [30], [31], [32], germline stem cells (GSCs) [33], [34] and dental pulp stem cells (DPNCs)[35] *et al*. Studies on these adult stem cells have shown many benefits. For example, expression of Oct4 alone has been found to be sufficient for the reprogramming of neural stem cells [32], which reduces the risk of exogenous gene integration. Furthermore, the feeder-independent system used to induce human immature DPNCs can decrease the potential variability caused by using feeder cells [35]. The common feature of these cells described above is that they are all adult stem cells, which may be appropriate cellular sources for reprogramming. West *et al* used BMSCs-derived piPSCs to produce chimeric pigs [11], although the results are controversial, their work suggested us that mesenchymal stem cells-derived iPSCs might be more available for the generation of porcine offspring. Here, we enriched the starting source with another type of mesenchymal stem cells, pADSCs, for reprogramming. Porcine ADSCs share many features with BMSCs, including immunotolerance, defined cell surface molecules, and potential for multilineage differentiation [36], [37]. However, obtaining these other adult stem cells (i.e., NSCs, GSCs, DPNCs and BMSCs) would be more difficult and potentially painful, while the acquisition of pADSCs from animals is relatively easy. A large amount of adipose tissue can be obtained from subcutaneous [38], interscapular [39] and inguinal [40] regions in a short period of time. In combination with their rapidly proliferate activity, large number of pADSCs could be acquired in short term. Altogether, pADSCs may be also an ideal reprogramming material for improving the quality of porcine iPSCs for the production of porcine offspring.

Another benefit of pADSCs for reprogramming is their efficient reprogramming. Studies on human and mouse had suggested that, the reprogramming process with ADSCs is more efficient and faster than with fibroblasts [1], [25], [41], which can be attributed to many factors. Mouse and human ADSCs possess high endogenous expression of cellular factors, such as FGF2, TGFß1, bronectin-1, vitronectin, activin A and Lif, which can support proliferation and maintenance of self-renewal of both autologous and heterologous pluripotent cells [25]. They also can express key pluripotency reprogramming genes at low levels, including Oct4, c-Myc, Klf4, Nanog, Rex-1 SSEA3, SSEA4, and Tra-1-60 [1], [25], [41]. However, present analysis of the key reprogramming factors suggested that the expression of Klf4 in pADSCs was only slightly higher than that in pEFs, indicating that it may not account for the high reprogramming efficiency of pADSCs. Therefore, the epigenetic status of the whole genome was investigated in further analysis. Modification of nucleic acid bases without changing the primary DNA base sequence is an important biological mechanism that regulates gene expression. The most common enzymatic DNA modification in eukaryotic cells is methylation of the 5-mC, which always leads to strong and heritable gene silencing [42], and therefore is essential for mammalian development and tissue differentiation [43], [44]. Recent evidence suggests that 5-mC can be converted to 5-hmC in mammalian DNA by MLL Partner TET1 [45], 5-hmC enrichment has been proposed to be involved in the demethylation and reactivation of genes and regulatory regions that are important for pluripotency, thus playing an important role in maintaining the stemness of ESCs and reprogramming of iPSCs [46], [47], [48]. The intrinsic higher level of 5-hmC and lower level of 5-mC in pADSCs than pEFs suggest a more flexible sel-renewal status, meanwhile that fewer roadblocks would be encountered during its reprogramming. The high level of demethylation in the Nanog promoter regions of pADSCs may also contribute to this process. Thus, the pattern of demethylation in pADSCs may be a key reason of their high efficiency in reprogramming.

Previous study has revealed the beneficial effect of naïve state PSCs on the production of filial animals [21], thus naïve-like state of piPSCs were established in our work. Before determining the naïve-like state of the two cell lines in the current study, their expression of genes related to a full reprogramming stage was confirmed. Subsequently, evidence for a naïve-like state was gathered by evaluating various characteristics of the NpiPSCs, including morphology, behavior in single-cell passaging, Lif-dependency, expression of specific genes and activation of both X chromosomes, which was confirmed by the demethylation of H3K27me3, as the important role of H3K27me3 in the initiation of X chromosome inactivation [49]. The evidence from single-cell passaging is particularly crucial, as it is highly important in genetic manipulation at the colony level and overcoming the high rate of transformation with routine passaging *in vitro* [50], which may severely block the development of reconstructed embryos during the production of iPSCs-derived offspring. Therefore, proper single cell-derived colonies may have to be re-selected. During the detection of multipotency related proteins, the expression of TRA-1-60 and TRA-1-81 were found to be very low in the NpiPSCs, which was obviously contradictory with a previous study on piPSCs [6], whereas it is also a characteristic of naïve state [51]. Jacob *et al* have summarized many features of the naïve state of mouse PSCs [51], some of which may be different from those of porcine PSCs such as the expression pattern of SSEA-1. However, these variations simply may be due to regulatory differences between species [6].

Since naïve PSCs are in a stage upstream of primed PSCs, obtaining naïve PSCs may be achieved by further reprogramming of primed PSCs [22], [52] or altering culture system [53]. This process mainly depends on the activation and repression of associated signaling pathways, including JAK/STAT3 [15], [54], [55], BMP/Smad [56], WNT/ß-catenin [55], [57] and MEK/ERK [55], [58]. Meanwhile, JAK/STAT3 signaling is known to be indispensable for the naïve state of PSCs, which is stimulated by Lif. In mice, Lif is sufficient and dominant over antagonistic cues for the establishment of its naïve pluripotency [5], [15]. However, this situation may not be universal for all the species. For example, many other inhibitors were found to be required for the establishment of authentic ESCs from rats with ground state [54]. Among these inhibitors, CHIR99021 and PD0325901 (2i), which activate the WNT signaling pathway and inhibit the ERK-mediated differentiation pathway, respectively, exhibit dominant roles in establishing and sustaining the naïve state of PSCs. Even though the 2i are not indispensable for the generation of naïve iPSCs in other species, such as mouse and human [15], [16], [53], they were found to efficiently enhance the reprogramming process of naïve-like iPSCs [15]. 2i-treated mouse ESCs exhibit lower expression of lineage-affiliated genes, reduced prevalence at promoters of the repressive histone modification H3K27me3, and fewer bivalent domains [18]. Furthermore, the combination of 2i with Lif can promote the ground state pluripotency in partially reprogrammed iPSCs [59] or convert the primed state of human ESCs into a naïve-like state [53]. One of the molecules, PD0325901, can stimulate the growth of true iPSCs and inhibit growth of non-iPSCs, which may facilitate the procurement of naïve-like iPSCs [60]. Another study on piPSCs has shown that the addition of 2i can pave the way for the generation of germline competent stem cells [61], and it is may be the reason for the complete reprogramming of our NpiPSCs when compared to previous work, which piPSCs failed to differentiate *in vivo* when cells were induced without 2i [21]. Consequently, the application of 2i may be more beneficial for the capture of porcine naïve iPSCs with fully reprogramming. Nevertheless, Lif-2i treatment functions best within a specified time frame, as Smith and colleagues have reported greater effectiveness of Lif-2i on NSCs reprogramming when chemical treatment was applied at day 5 rather than day 3 [19]. This observation can be attributed to the fact that the MEK signaling pathway, which is inhibited by PD0325901, is required for somatic cell survival [62], and is consistent with our studies.

Human and mouse adipose-derived cells are known to support feeder-independent induction of pluripotent stem cells [25]. Accordingly, we have developed a feeder-independent and serum-free system to generate naïve-like piPSCs. Use of a feeder-independent induction system can promote the biosafety of naïve-like piPSCs by avoiding the contamination of xenogenic cells, which may increase the risk of immunorejection for recipient animals in transplantation research [63], [64]. Without feeder cells, some basic studies will be more convenient to perform, as the unknown cell-to-cell interactions may interfere with experimental processes under investigation [65]. However, feeders are still essential for subsequent passages in present. Therefore, feeders from pADSCs could be an alternative option to help avoid xenogenic contamination [66], [67], while further research is needed in this area. Nevertheless, serum-free system is necessary for the maintenance of a naïve state. A previous study has shown that mouse ESCs grown in serum exhibit greater heterogeneity in morphology and expression of pluripotency factors than cultured in defined medium with 2i [18]. The unknown components in serum can activate the ERK pathway, ultimately resulting in the loss of the naïve state [68], as well as decreased Rex1 expression and core pluripotency factors such as Nanog and Klf4 in some cells [18].Thus, the quality and safety of naïve-like piPSCs may be improved by feeder-independent and serum-free conditions.

ESCs have displayed the immunologic tolerance during syngeneic transplantation [69], while studies on the immunogenicity of iPSCs are controversial. Araki *et al* and Guha *et al* have demonstrated that cells originating from chimeric mice and iPSCs can be immune-tolerated by syngeneic recipients [70], [71]. Whereas contradictory result was described by Zhao *et al* [69]. Many factors can impact the immunogenicity of iPSCs, including reprogramming method [69], [72], stage of differentiation [73] and microevironment of iPSCs [70], [74]. The major antigens mediating immune rejection is the major histocompatibility antigen (MHC) family of proteins (including MHC I, MHC II and MHC III), and MHC I is one of main molecules causing immune responses [75], [76], [77]. Naïve-like piPSCs derived in our system were negative for MHC I, implying low immunogenicity. Furthermore, the cellular origin of iPSCs may influence their molecular and functional properties [78], low expression of MHC I is also the instinct characteristic of ADSCs [29], which may facilitate the loss of MHC I during reprogramming of the naïve-like piPSCs.

Fan and colleagues attempted to obtain piPSCs with lentiviral and retrovirus vectors [4], and found that embryos derived from piPSCs obtained with the retrovirus method stopped developing *in vivo*, while those obtained with lentiviral method would develop to birth. Retrovirus could infect cells in diving phase and non-diving phase, as their random integration in genome, it may activate the cancer-related genes. While the SV40T antigen in the lentiviral vector makes the reprogramming process more efficient than that with a retrovirus vector [79]. Even though the risk also exists for lentiviral, it is much safer than retrovirus [80]. In their further study, they found that the incomplete silencing of exogenous genes can seriously impair the development of embryos *in vivo* [4], which is similar to the finding of a previous work on naïve-like piPSCs [21]. And this might be caused by the immunogenicity of exogenous reprogramming factors [72]. In our study, naïve-like piPSCs generated with a lentiviral vector were exerted as donor cells to make reconstructed embryos. The quality of reconstructed embryos from naïve-like piPSCs was significantly improved with the estimation of whole blastomere number. Meanwhile, drug-inducible system were also used in present study, it could regulate the expression and silencing of exogenous genes efficiently, which may avoid the risk of immunorejection during *in vivo* development, despite further study is needed.

In conclusion, a key point affecting the production of piPSCs-derived offspring is the quality of these cells. In the present work, we utilized pADSCs as the source material to demonstrate an efficient reprogramming process. The drug-inducible system employed for the reprogramming method facilitated the silencing of exogenous genes, which may be helpful for reducing the risk of immune rejection and gene mutation of embryos developing *in vivo*. Meanwhile, pADSCs were shown to support the generation of piPSCs under feeder-independent and serum-free conditions, thereby avoiding unknown factors in feeders and serum which are potentially unfavorable for the cellular epigenetic reprogramming. Naïve-like piPSCs were finally obtained under Lif-2i treatment and subsequent culture system, which would improve the quality of reconstructed embryos and ease the genome modification process in future application. Consequently, through careful selection and evaluation of the cellular source material, as well as the reprogramming method, environment and state of the cells, we have improved the quality of porcine iPSCs, which may be suitable for the production of porcine offspring through nuclear transfer approach.

## Supporting Information

Figure S1 **The growth characteristic of pADSCs at passage 3.** Initial concentration of 10,000 cells/well was seeded in a 24-well plate, three wells per plate were counted every 24 h after trypsinization.
(TIF)

Figure S2 **The whole genomic methylation and demethylation level of pADSCs and pEFs.** The methylation level of pADSCs (A, scale bar = 100 µm) and pEFs (B, scale bar = 100 µm) were evaluated by immunostaining, which with antibody was directed to 5-mC. And the demethylation level of pADSCs (C, scale bar = 100 µm) and pEFs (D, scale bar = 100 µm) were estimated by antibody directed to 5-hmC.
(TIF)

Table S1 **Porcine Primers for Real-time PCR.**
(DOC)

Table S2 **Porcine Primers for Reverse transcription PCR.**
(DOC)

Table S3 **Porcine Primers for semi-nested PCR.**
(DOC)
