# Supplementary figures and images for: Efficient Reprogramming of Naïve-Like Induced Pluripotent Stem Cells from Porcine Adipose-Derived Stem Cells with a Feeder-Independent and Serum-Free System
Source: PLoS One. 2014 Jan 20;9(1):e85089. doi: 10.1371/journal.pone.0085089 (PMC3896366; doi:10.1371/journal.pone.0085089)

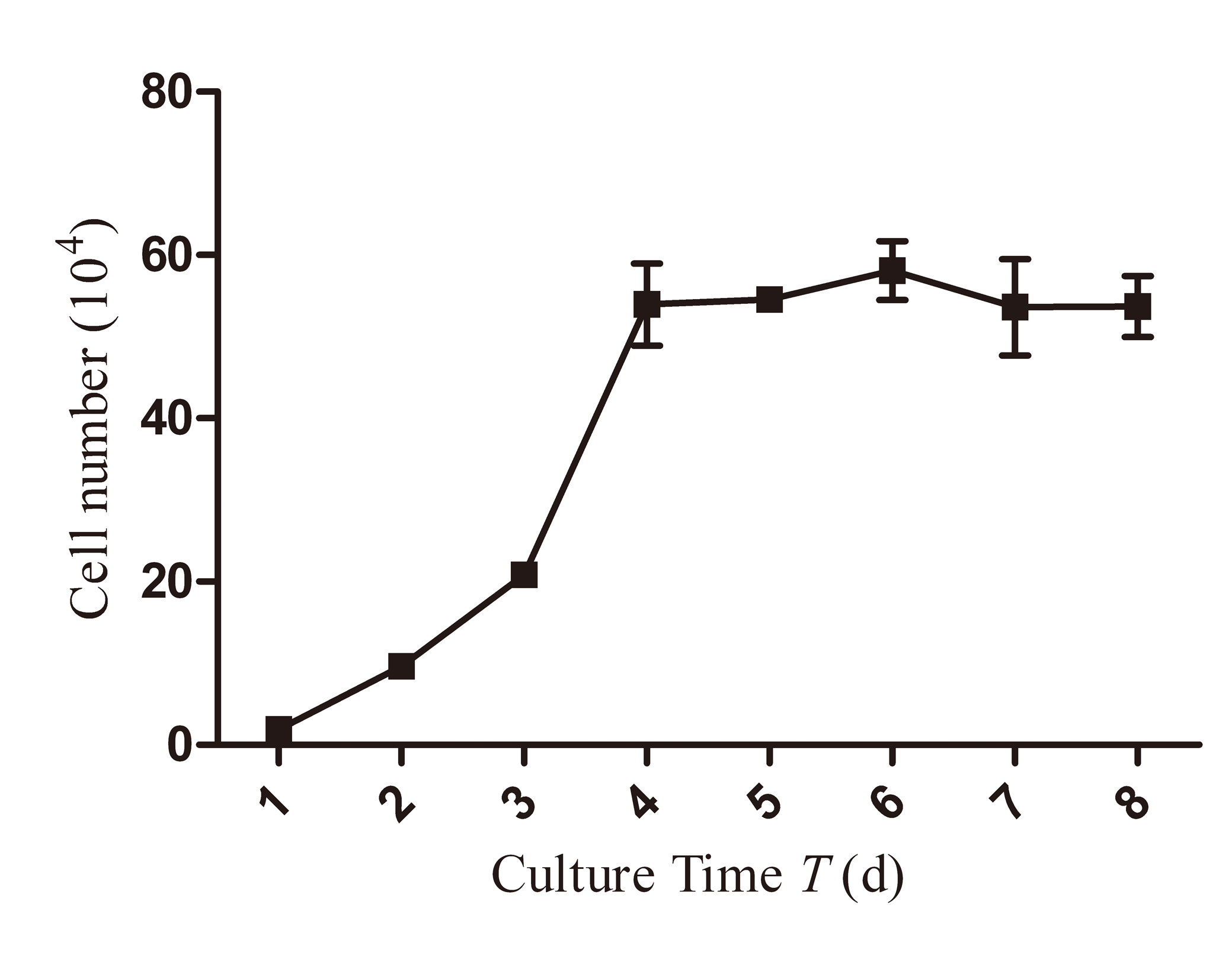

Supplement: Figure S1 — The growth characteristic of pADSCs at passage 3. Initial concentration of 10,000 cells/well was seeded in a 24-well plate, three wells per plate were counted every 24 h after trypsinization. (TIF) [file pone.0085089.s001.tif]

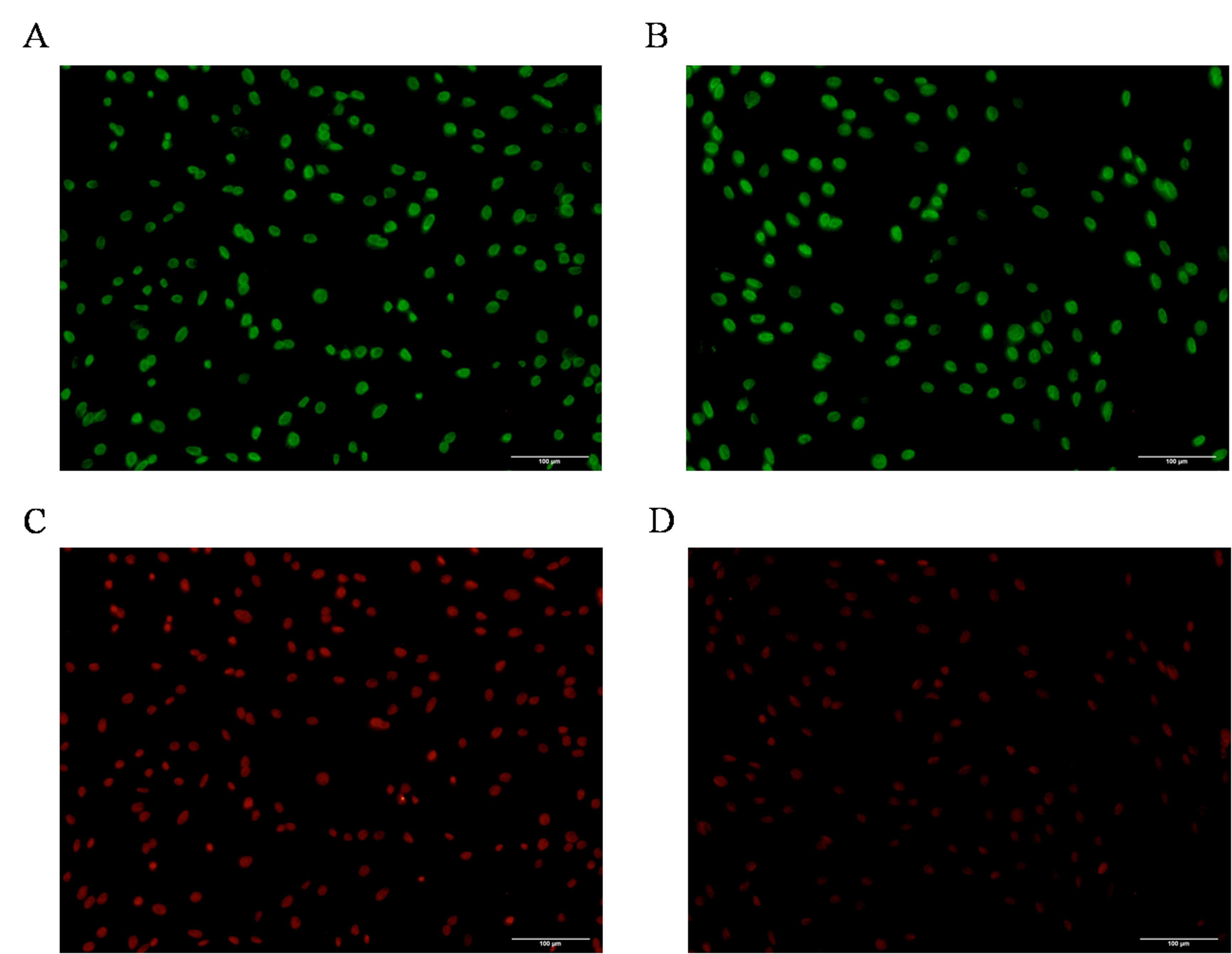

Supplement: Figure S2 — The whole genomic methylation and demethylation level of pADSCs and pEFs. The methylation level of pADSCs (A, scale bar = 100 µm) and pEFs (B, scale bar = 100 µm) were evaluated by immunostaining, which with antibody was directed to 5-mC. And the demethylation level of pADSCs (C, scale bar = 100 µm) and pEFs (D, scale bar = 100 µm) were estimated by antibody directed to 5-hmC. (TIF) [file pone.0085089.s002.tif]
