# Supplementary material for: Efficient Reprogramming of Naïve-Like Induced Pluripotent Stem Cells from Porcine Adipose-Derived Stem Cells with a Feeder-Independent and Serum-Free System
Source: PLoS One. 2014 Jan 20;9(1):e85089. doi: 10.1371/journal.pone.0085089 (PMC3896366; doi:10.1371/journal.pone.0085089)
Supplement: Table S1 — Porcine Primers for Real-time PCR. (DOC) [file pone.0085089.s003.doc]

| Gene Name | Sequence (5' to 3') | |
| --- | --- | --- |
| Endo Oct4 | F | CAAACTGAGGTGCCTGCCCTTC |
|  | R | ATTGAACTTCACCTTCCCTCCAACC |
| Endo Sox2 | F | GTTCCATGGGCTCAGTGGTCAAG |
|  | R | AAGCGTACCGGGTTTTTCTCCATAC |
| Endo Klf4 | F | ACACCTGCGAACCCACACA |
|  | R | GCGGTAGTGCCTGGTCAGTT |
| Endo c-Myc | F | CTGAAGACAGGCCGAGTGATG |
|  | R | CCACAGGCTGATTTCTTTCTCA |
| Nanog | F | AGCCCCAGCTCCAGTTTCAGC |
|  | R | AATGATCGTCACATATCTTCAGGCTGTA |
| Lin28 | F | CAGAGTAAGCTGCACATGGAGG |
|  | R | GTAGGCTGGCTTTCCCTGTG |
| Dnmt3b | F | AACCCAACAAAGCAACCAG |
|  | R | CCGACCACAGGATAAACAG |
| Tert | F | CTGGAGGTGCACTGCGACTAT |
|  | R | GCCCTGGTTGAAGGTGAGACT |
| Esrrb | F | AGGGAGCTCGTGGTCATCAT |
|  | R | CCCCAGAGAGAGGTTGGAGAA |
| Utf1 | F | CCGCGGGCCCGACCTCACG |
|  | R | GAACGCCGCCCTCCTGCAGACCTT |
| Dppa5 | F | GATGCTCCAGTCTATGGCAGAGT |
|  | R | GTGAATTCATGGCTTCCTCAAGTC |
| Stella | F | TTAATCCAACCCGGACTCAG |
|  | R | TGGTTGAGGTGGATATTCTGG |
| Eras | F | CTGGTGATGGTGTGCTGGGCGTCT |
|  | R | CACGGCTTTCTGGTGTCGGGTCTT |
| MHC Class I | F | CCTCTTCCTGCTGCTGTCG |
|  | R | AGCGTGTCCTTCCCCATCT |
| Gapdh | F | GCAATGCCTCCTGTACCACC |
|  | R | TCACGCCACAGTTTCCCAG |
