# Supplementary material for: Efficient Reprogramming of Naïve-Like Induced Pluripotent Stem Cells from Porcine Adipose-Derived Stem Cells with a Feeder-Independent and Serum-Free System
Source: PLoS One. 2014 Jan 20;9(1):e85089. doi: 10.1371/journal.pone.0085089 (PMC3896366; doi:10.1371/journal.pone.0085089)
Supplement: Table S2 — Porcine Primers for Reverse transcription PCR. (DOC) [file pone.0085089.s004.doc]

| Gene Name | Sequence (5' to 3') | |
| --- | --- | --- |
| Neurod | F | GACTTGCGTTCAGGCAAAAGC |
|  | R | GGGCGACTGGTAAGAGTAGG |
| Sox9 | F | AGAAGGAGAGCGAAGAGGACAA |
|  | R | CGTCCAGTCGTAGCCCTTGA |
| Pdx1 | F | GCCTTTCCCGTGGATGAAG |
|  | R | CGGTTTTGGAACCAGATTTTG |
| Exo Oct4 | F | AGAAGGATGTGGTCCGAGTGTG |
|  | R | CAGAGTGGTGACAGAGACAGGG |
| Exo Sox2 | F | TCTTGGCTCCATGGGTTCGG |
|  | R | AGTGCTGGGACATGTGAAGTCTG |
| Exo c-Myc | F | TCAAGAGGCGAACACACAAC |
|  | R | GGCCTTTTCATTGTTTTCCA |
| Exo Klf4 | F | GGCGGTCTCTTCGTGCACCCACT |
|  | R | CCCTGCTGCTCAGCACTTCCTCAAG |
| Gapdh | F | GCAATGCCTCCTGTACCACC |
|  | R | TCACGCCACAGTTTCCCAG |
