# Supplementary material for: Efficient Reprogramming of Naïve-Like Induced Pluripotent Stem Cells from Porcine Adipose-Derived Stem Cells with a Feeder-Independent and Serum-Free System
Source: PLoS One. 2014 Jan 20;9(1):e85089. doi: 10.1371/journal.pone.0085089 (PMC3896366; doi:10.1371/journal.pone.0085089)
Supplement: Table S3 — Porcine Primers for semi-nested PCR. (DOC) [file pone.0085089.s005.doc]

| Gene Name | Sequence (5' to 3') | Region (ATG +1) |
| --- | --- | --- |
| Nanog -OF | GGATTAGATGGTTTTATAGGTG | -1624~-1602 |
| Nanog -IF | AGGTTGGGTTTATTTTAGGT | -1349~ -1330 |
| Nanog -R | ATATTCCCTCTATACCCACTTAAC | -1148~ -1162 |
